# Expression of the thymidine phosphorylase gene in epithelial ovarian cancer

**DOI:** 10.1038/sj.bjc.6690294

**Published:** 1999-04

**Authors:** K Hata, T Kamikawa, S Arao, H Tashiro, H Katabuchi, H Okamura, R Fujiwaki, K Miyazaki, M Fukumoto

**Affiliations:** Department of Obstetrics and Gynecology, Shimane Medical University, Izumo, 693-8501, Japan; Department of Pathology and Biology of Disease, Graduate School of Medicine, Kyoto University, Kyoto, 606-8315, Japan; Department of Obstetrics and Gynecology, School of Medicine, Kumamoto, 860-0811, Japan; Department of Pathology, Institute of Development, Aging and Cancer, 4–1 Seiryomachi Aubu-Ku, Sendai, 980-8575, Japan

**Keywords:** thymidine phosphorylase, gene expression, reverse transcriptase polymerase chain reaction, epithelial ovarian cancer

## Abstract

Thymidine phosphorylase (TP) is associated with angiogenesis and the progression of solid tumours. High intracellular levels of this enzyme indicate increased chemosensitivity to pyrimidine antimetabolites. *TP* gene expression in 56 cases of epithelial ovarian cancer (27 of serous, 10 mucinous, 12 endometrioid, five clear cell and two undifferentiated) were analysed by polymerase chain reaction of RNA after reverse transcription. These included eight of low malignant potential. Twenty were stage I, four stage II, 27 stage III and five stage IV. The level of *TP* gene expression was presented by the relative yield of the *TP* gene to the *β2-microglobulin* gene. *TP* gene expression ranged from 0.19 to 5.38 (median 0.93). The value of *TP* gene expression in stage III–IV was significantly higher than that of *TP* gene expression in stage I–II (*P* = 0.0005). Histological grade significantly associated with *TP* gene expression (*P* = 0.008), but histological subtype did not (*P* = 0.166). A follow-up study of 34 cases after complete resection of the primary tumours by surgical operation was performed. *TP* gene expression of the cases with recurrence showed significantly higher levels compared to cases without recurrence (*P* = 0.049). Survival data were available for 47 of the 56 patients. The prognosis of the patients with high *TP* gene expression (equal to, or greater than, median) was to be significantly worse than patients with low *TP* gene expression (less than median) (*P* = 0.021). The *TP* gene expression level may play one of the key roles in the biology of ovarian epithelial cancer and define a more aggressive tumour phenotype. A new therapeutic intervention mediated by TP protein activity is anticipated. © 1999 Cancer Research Campaign


*British Journal of Cancer* (1999) **79**(11/12), 1848–1854
© 1999 Cancer Research Campaign
Article no. bjoc.1998.0294

### Expression of the thymidine phosphorylase gene in epithelial ovarian cancer

**K Hata[1], T Kamikawa[2], S Arao[3], H Tashiro[3], H Katabuchi[3], H Okamura[3], R Fujiwaki[1], K Miyazaki[1] and M Fukumoto[4]**

[1]Department of Obstetrics and Gynecology, Shimane Medical University, Izumo 693-8501, Japan; [2]Department of Pathology and Biology of Disease, Graduate School of Medicine, Kyoto University, Kyoto 606-8315, Japan; [3]Department of Obstetrics and Gynecology, School of Medicine, Kumamoto 860-0811, Japan; [4]Department of Pathology, Institute of Development, Aging and Cancer, Tohoku University, 4–1 Seiryomachi Aubu-Ku, Sendai 980-8575, Japan

**Summary** Thymidine phosphorylase (TP) is associated with angiogenesis and the progression of solid tumours. High intracellular levels of this enzyme indicate increased chemosensitivity to pyrimidine antimetabolites. *TP* gene expression in 56 cases of epithelial ovarian cancer (27 of serous, 10 mucinous, 12 endometrioid, five clear cell and two undifferentiated) were analysed by polymerase chain reaction of RNA after reverse transcription. These included eight of low malignant potential. Twenty were stage I, four stage II, 27 stage III and five stage IV. The level of *TP* gene expression was presented by the relative yield of the *TP* gene to the *β2-microglobulin* gene. *TP* gene expression ranged from 0.19 to 5.38 (median 0.93). The value of *TP* gene expression in stage III–IV was significantly higher than that of *TP* gene expression in stage I–II ($P = 0.0005$). Histological grade significantly associated with *TP* gene expression ($P = 0.008$), but histological subtype did not ($P = 0.166$). A follow-up study of 34 cases after complete resection of the primary tumours by surgical operation was performed. *TP* gene expression of the cases with recurrence showed significantly higher levels compared to cases without recurrence ($P = 0.049$). Survival data were available for 47 of the 56 patients. The prognosis of the patients with high *TP* gene expression (equal to, or greater than, median) was to be significantly worse than patients with low *TP* gene expression (less than median) ($P = 0.021$). The *TP* gene expression level may play one of the key roles in the biology of ovarian epithelial cancer and define a more aggressive tumour phenotype. A new therapeutic intervention mediated by TP protein activity is anticipated.

**Keywords:** thymidine phosphorylase; gene expression; reverse transcriptase polymerase chain reaction; epithelial ovarian cancer

Thymidine phosphorylase (TP) is an enzyme which catalyses two reactions: (i) the reversible phosphorylation of thymidine to thymine and 2′-deoxy-*D*-ribose-1-phosphate, and (ii) deoxyribosyl transfer between pyrimidines. Overexpression of the enzyme has been associated with the development of various cancers (Pauly et al, 1997; Yoshimura et al, 1990). The structure of human TP is identical (at least in part) to that of platelet-derived endothelial cell growth factor (PD-ECGF) (Furukawa et al, 1992; Moghaddam et al, 1992). TP–PD-ECGF has been shown to possess angiogenic activity in vivo (Ishikawa et al, 1989), and chemotactic activity in vitro (Haraguchi et al, 1994). The analysis of mRNA for putative angiogenic factors in ovarian malignancies revealed the overexpression of TP–PD-ECGF compared with tissue from benign tumours and the normal ovary (Reynolds et al, 1994). Subsequently, the concentration of TP in ovarian tumour tissue was shown to be positively correlated with the peak systolic velocity measured by pulsed-Doppler ultrasound (Hata et al, 1997) which is an indicator of disordered angiogenesis (Hata et al, 1995).

Although progress has been made in establishing operative staging of ovarian cancer and defined basic principles of therapy, there has not been a dramatic change in survival rates since the 1960s (Griffiths and Parker, 1986). The early stages of malignant growth in the ovary do not usually produce symptoms, and late diagnosis and ineffective treatments are probably the main reasons for the poor prognosis. Despite extensive research, the mortality does not seem to be decreasing (Bourne et al, 1993). However, even in patients presenting with early disease, an extensive surgical treatment does not guarantee cure (Scholl et al, 1994). Therefore, new variables correlating with the malignant potential of the cancer cells would be clinically valuable in adjuvant therapy for individual patients in early stage ovarian cancer. Biological factors that regulate the growth of this disease are poorly defined. Recently, there have been studies on the correlation between the expression of TP by immunohistochemical analysis, and invasiveness and progression of tumours in breast (Fox et al, 1996), lung (Giatromanolaki et al, 1997), gastric (Maeda et al, 1996), colon (Takebayashi et al, 1996) and bladder cancers (O'Brien et al, 1996). Potential for its clinical relevance has been reported; however, the results differ among each cancer (Fox et al, 1996; Maeda et al, 1996; O'Brien et al, 1996; Takebayashi et al, 1996; Giatromanolaki et al, 1997).

In the present study, we have examined mRNA expression of the TP using reverse transcriptase polymerase chain reaction (RT-PCR) in 56 epithelial ovarian cancers. The expression of this enzyme has been related to clinical and pathological parameters to evaluate further the role of TP in epithelial ovarian cancer.

*Received 20 May 1998*
*Revised 10 August 1998*
*Accepted 21 August 1998*

*Correspondence to:* M Fukumoto

### MATERIALS AND METHODS

### Patients

A total of 56 patients (aged 19–76 years, mean 50 years) with histologically confirmed primary epithelial ovarian cancer were studied (Table 1). No patient received any therapy before surgery. The patients were staged according to criteria recommended by the International Federation of Obstetricians and Gynecologists (FIGO) criteria (1987). The staging system defined by FIGO assumes that an adequate staging operation has been performed (Cannistra, 1993). The staging operation included collection of ascites or peritoneal washing from the pelvis, gutters and diaphragms for cytological studies; total abdominal hysterectomy with bilateral salpingoophorectomy; infracolic omentectomy and appendectomy; selective pelvic and para-aortic lymphadenectomy; and debulking of all gross diseases. If obvious macroscopic tumour was not present, the following were performed: biopsy of any lesion suspect for tumour metastasis or any adhesion adjacent to the primary tumour; blind biopsy of bladder peritoneum and cul-de-sac, right and left paracolic gutter and pelvic side walls; biopsy or smear of right hemidiaphragm. Survival data were available for 47 of the 56 patients (median 36 months, range 2–120 months). Of these, 45 patients received cisplatin-containing regimens. Two stage I tumours of low malignant potential received no further treatment after surgery.

### Tissue specimen and RNA preparation

Fresh surgical specimens from all patients were obtained, and the tissues for investigation were prepared carefully under a dissecting microscope to eliminate inappropriate components. Moreover, one stage I epithelial ovarian cancer (serous cystadenocarcinoma) with primary colorectal cancer that resulted in death 2 months after operation and benign ovarian tumour tissues (serous cystadenoma, fibroma) were dissected. The tissue samples were stored at –80°C for subsequent analysis. Normal liver tissues were used as positive control for *TP* gene expression. As negative control for *TP* gene expression, breast carcinoma cell line MCF-7 was kindly provided by Dr Akira Yamauchi.

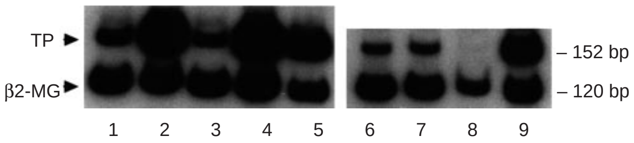

**Figure 1** The representative thymidine phosphorylase (*TP*) gene expression by RT-PCR (β2-MG: β2-microglobulin, lane 1; ovarian cancer stage I, lane 2; ovarian cancer (serous cystadenocarcinoma) stage I with primary colorectal cancer which resulted in death 2 months after operation, lane 3; ovarian cancer stage II, lane 4; ovarian cancer stage III, lane 5; ovarian cancer stage IV, lanes 6 and 7; benign ovarian tumour, lane 8; MCF-7, lane 9; normal liver)

**Table 1** Thymidine phosphorylase gene expression in each ovarian cancer

| Case | Age (years) | Stage | Histology | Histological grade[a] | *TP* gene expression | Case | Age (years) | Stage | Histology | Histological grade[a] | *TP* gene expression |
|---|---|---|---|---|---|---|---|---|---|---|---|
| 1 | 28 | I | Serous | 1 | 1.33 | 29 | 61 | I | Mucinous | 1 | 0.41 |
| 2 | 56 | I | Serous | 1 | 0.25 | 30 | 26 | I | Mucinous | 1 | 0.40 |
| 3 | 42 | I | Serous | 1 | 0.94 | 31 | 53 | I | Mucinous | 2 | 1.41 |
| 4 | 54 | II | Serous | 2 | 0.20 | 32 | 58 | I | Mucinous | 1 | 0.61 |
| 5 | 58 | III | Serous | 3 | 2.08 | 33 | 47 | I | Mucinous | 4 | 1.79 |
| 6 | 48 | III | Serous | 3 | 0.71 | 34 | 38 | I | Mucinous | 1 | 0.44 |
| 7 | 58 | III | Serous | 4 | 2.48 | 35 | 46 | III | Mucinous | 3 | 0.37 |
| 8 | 58 | III | Serous | 3 | 1.30 | 36 | 42 | III | Mucinous | 2 | 0.36 |
| 9 | 63 | III | Serous | 4 | 5.38 | 37 | 47 | IV | Mucinous | 4 | 3.21 |
| 10 | 24 | III | Serous | 2 | 0.55 | 38 | 60 | I | Endometrioid | 3 | 0.25 |
| 11 | 40 | III | Serous | 3 | 2.46 | 39 | 45 | I | Endometrioid | 3 | 0.24 |
| 12 | 61 | III | Serous | 2 | 0.82 | 40 | 51 | I | Endometrioid | 4 | 0.44 |
| 13 | 66 | III | Serous | 3 | 4.47 | 41 | 60 | I | Endometrioid | 2 | 0.42 |
| 14 | 56 | III | Serous | ND | 0.35 | 42 | 43 | I | Endometrioid | 3 | 1.65 |
| 15 | 63 | III | Serous | 2 | 0.28 | 43 | 38 | I | Endometrioid | 4 | 1.14 |
| 16 | 46 | III | Serous | 4 | 0.30 | 44 | 70 | II | Endometrioid | 3 | 0.22 |
| 17 | 54 | III | Serous | 2 | 0.19 | 45 | 54 | II | Endometrioid | 3 | 1.08 |
| 18 | 57 | III | Serous | 4 | 2.08 | 46 | 46 | III | Endometrioid | 4 | 3.37 |
| 19 | 67 | III | Serous | 4 | 0.80 | 47 | 49 | III | Endometrioid | 4 | 0.25 |
| 20 | 48 | III | Serous | 3 | 5.10 | 48 | 37 | III | Endometrioid | 3 | 1.44 |
| 21 | 53 | III | Serous | 3 | 2.10 | 49 | 45 | III | Endometrioid | 3 | 1.83 |
| 22 | 49 | III | Serous | 2 | 1.10 | 50 | 52 | I | Clear cell | 4 | 1.21 |
| 23 | 55 | III | Serous | 3 | 2.80 | 51 | 47 | I | Clear cell | 3 | 0.56 |
| 24 | 30 | IV | Serous | 3 | 0.92 | 52 | 40 | I | Clear cell | 2 | 0.48 |
| 25 | 62 | IV | Serous | 4 | 0.97 | 53 | 60 | I | Clear cell | 3 | 2.80 |
| 26 | 33 | IV | Serous | 4 | 2.82 | 54 | 47 | II | Clear cell | ND | 0.65 |
| 27 | 19 | IV | Serous | 3 | 2.73 | 55 | 48 | III | Undifferentiated | 4 | 4.70 |
| 28 | 55 | I | Mucinous | 1 | 0.29 | 56 | 76 | III | Undifferentiated | 4 | 0.71 |

[a]1, low malignant potential; 2, well differentiated; 3, moderately differentiated; 4, poorly differentiated; ND, not determined

*© Cancer Research Campaign 1999*

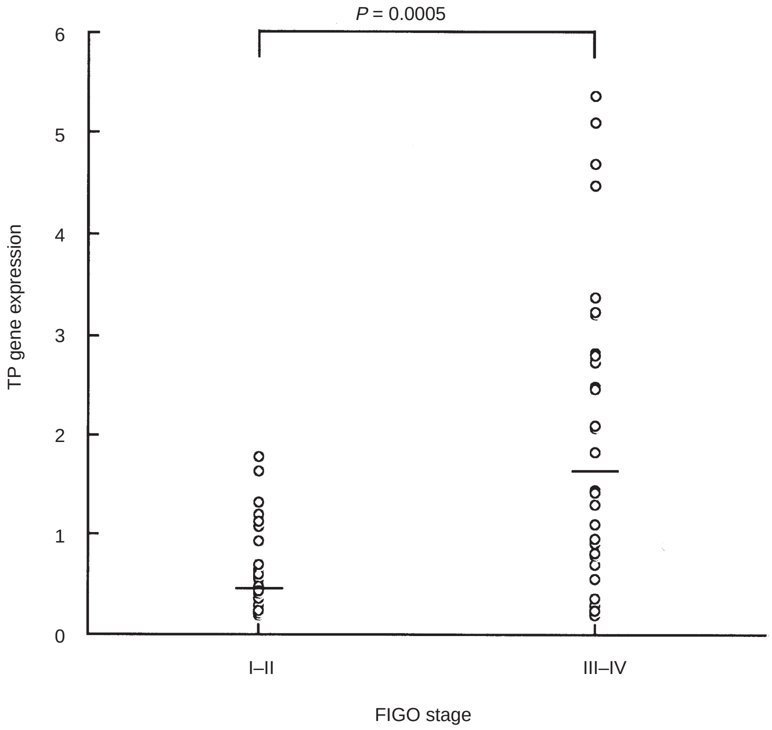

**Figure 2** Thymidine phosphorylase (*TP*) gene expression in stage I–II and III–IV. Horizontal line indicates the median value

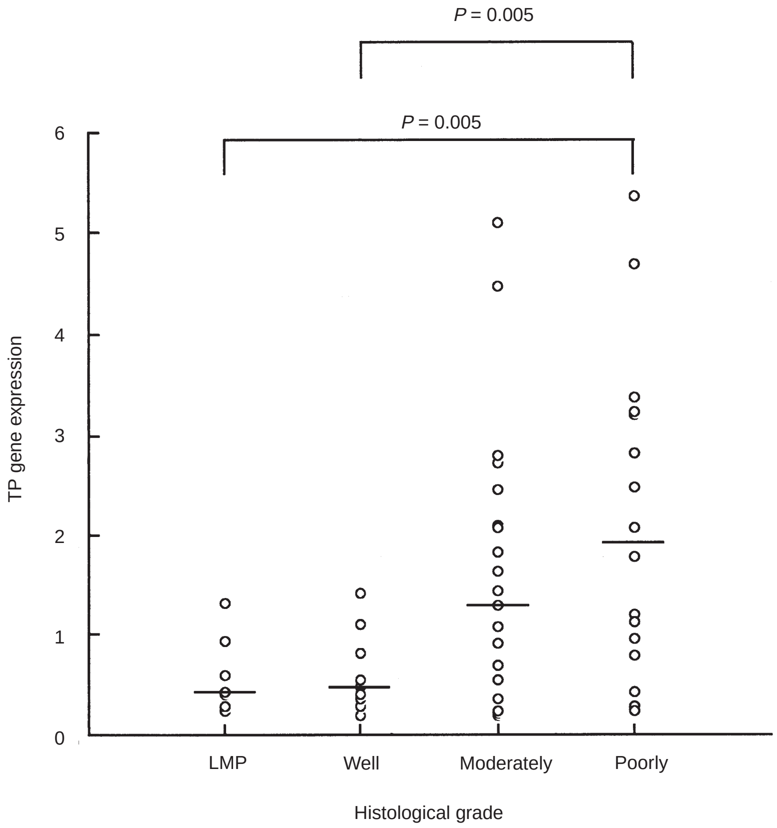

**Figure 3** Thymidine phosphorylase (*TP*) gene expression in relation to histological grades (1, low malignant potential; 2, well differentiated; 3, moderately differentiated; 4, poorly defferentiated). Horizontal line indicates the median value

*© Cancer Research Campaign 1999*

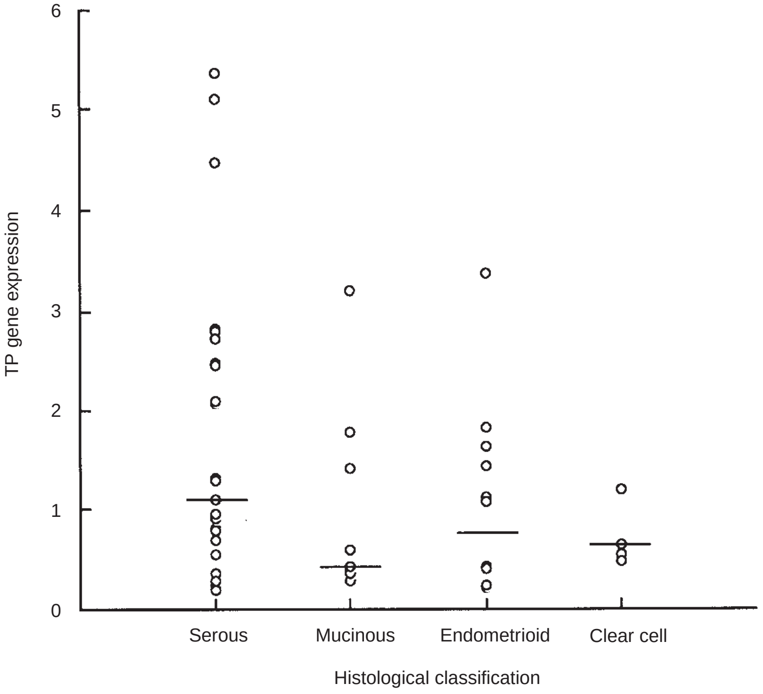

**Figure 4** Thymidine phosphorylase (*TP*) gene expression in relation to histological subtypes. Horizontal line indicates the median value

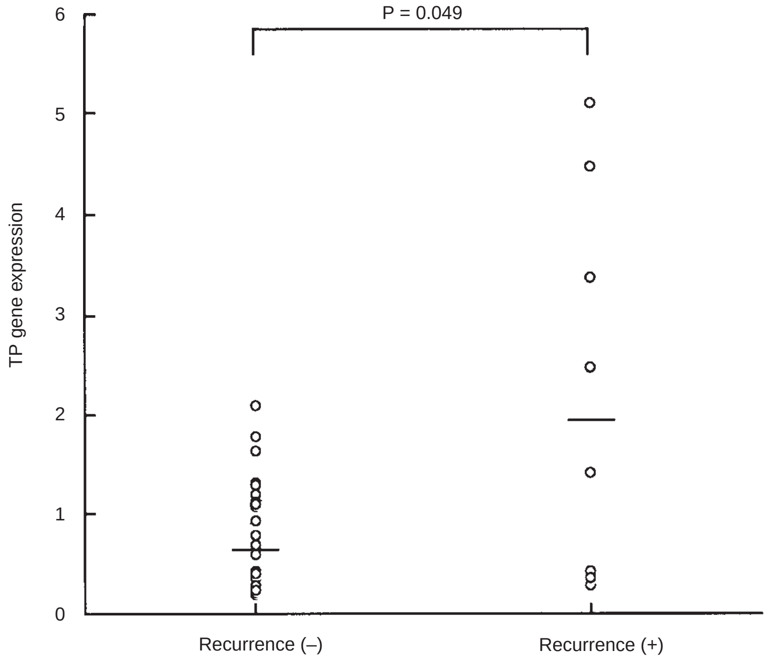

**Figure 5** Thymidine phosphorylase (*TP*) gene expression in patients with recurrence and without recurrence after complete resection. Horizontal line indicates the median value

### RT-PCR

RT-PCR for determination of *TP* gene expression was performed according to the method previously described (Arao et al, 1994). Briefly, complementary DNA (cDNA) was prepared by random priming from 500 ng of total RNA using a First-Strand cDNA Synthesis kit (Pharmacia-LKB, Uppsala, Sweden). Pairs of oligonucleotide primers for PCR were designed to insert an intron in the corresponding genomic sequence to eliminate amplification from genomic DNA. Primers for *TP* gene amplification is TGGCTCAGTCGGGACAGCAG (upstream) and TCCGCTGATCATTGGCACCT (downstream), and the PCR product is 152 bp (Hagiwara et al, 1991). The PCR was carried out in a Thermal

© Cancer Research Campaign 1999

Cycler (Perkin-Elmer Cetus, Northwalk, CT, USA) with a mixture consisting of cDNA derived from 5 ng of RNA, 10 pmol of upstream and downstream primers for the sequences of the *TP* gene and 5 pmol of primers for the *β2-microglobulin* (*β2-MG*) gene, 200 μmol of deoxynucleotide triphosphate, 37 kBq of [α-$^{32}$P] dCTP, and 0.1 unit of *Taq* DNA polymerase with reaction buffer (Life Technologies, Rockville, MD, USA) in a final volume of 10 μl. The condition for PCR was denaturation at 94°C for 1 min, annealing at 58°C for 1 min and extension at 72°C for 1 min. Thirty cycles of PCR were performed for each specimen, and the products were separated on 9% polyacrylamide gels. Then radioactivity was determined by BAS 2000 Bioimage Analyzer (Fujix, Tokyo, Japan). The TP expression was presented by the relative yield of the *TP* gene to that of the *β2-MG* gene.

### Histological grading

Histologically we classified tumours into four grades including a non-invasive grade as previously described (Arao et al, 1994). The brief concept of grading used in the present study is as follows. A tumour of grade 1 is low malignant potential (LMP), which is non-invasive to surrounding tissues. Tumours of grade 2–4 correspond to well differentiated, moderately differentiated and poorly differentiated carcinoma respectively.

### Statistical analysis

Mann–Whitney *U*-test and Kruskal–Wallis one-way analysis of variance by ranks were used as appropriate for the evaluation of significant differences between end-points. Survival curves were calculated using the Kaplan–Meier method and analysed by the log-rank test. Factors significantly related to survival in the log-rank test were analysed by Cox's proportional hazard model with a stepwise regression analysis (Cox, 1972). A *P*-value < 0.05 was considered to be statistically significant.

### RESULTS

### RT-PCR and *TP* gene expression

To determine the number of PCR cycles appropriate for quantification, PCR was performed from 20 to 50 cycles at increase of 5 cycles. The expression ratios of *TP* to *β2-MG* were reasonably constant from 25 to 40 cycles (data not shown). Therefore, in the subsequent experiments, the values at 30 PCR cycles were defined as the expression of target genes. The representative profile of *TP* gene expression by RT-PCR is shown in Figure 1.

### TP gene expression and clinicopathological features

*TP* gene expression in ovarian cancer tissues is summarized in Table 1 (median 0.93, range 0.19–5.38). The numbers of gene expression are mean values from at least three independent RT-PCR experiments. The value of *TP* gene expression in stage III–IV (median 1.64, range 0.19–5.38) was significantly higher than that in stage I–II (median 0.46, range 0.20–1.79) ($P = 0.0005$) (Figure 2). Histological grade significantly associated with *TP* gene expression ($P = 0.008$) (Figure 3); however, histological subtype did not ($P = 0.166$) (Figure 4).

### TP gene expression and prognosis

A follow-up study of 34 cases after complete resection of the primary tumours by surgical operation. *TP* gene expression of the eight cases with recurrence (median 1.95, range 0.28–5.10) showed significantly higher levels compared to that of 26 cases without recurrence (median 0.66, range 0.20–2.10) (Figure 5). Survival data were available for 47 of the 56 patients. As shown in Figure 6, we found the prognosis of the patients with high *TP* gene expression equal to, or greater than, median to be significantly worse than that of those with low *TP* gene expression less than median by log-rank test ($P = 0.021$). Moreover, FIGO stage (III–IV) ($P = 0.001$), residual disease (> 2 cm) ($P = 0.006$) and histological grade (poorly differentiated) ($P = 0.048$) were found to be significantly associated with worse prognosis in univariate analysis (Table 2). Multivariate analysis of survival using Cox's proportional hazard model revealed that FIGO stage (III–IV) is an independent prognostic factor ($P = 0.041$) (Table 3).

### DISCUSSION

In spite of significant advances in surgery and the use of new, more effective chemotherapeutic regimens, the overall 5-year survival of patients with ovarian cancer is about 30%. Multivariate analysis of 21 240 cases of primary ovarian epithelial cancer showed that stage, histology, grade, age, the presence of ascites, lymph node status and race are all predictors of survival (Kosary, 1994). Identification of new prognostic factors might be of value in directing therapy and intensifying follow-up for a select group of patients.

The advent and development of PCR, a highly sensitive and efficient method of amplifying specific DNA segments present at low concentrations, provides an alternative approach for estimating the relative gene expression in small amount of tissues (Eisenstein, 1990). By amplifying cDNA reverse transcribed from RNA, the PCR can be used to measure quantitative expression of specific genes in tumour cells (Arao et al, 1994). While gene expression is not a direct measure of enzyme activity, Mimori et al (1997) reported that there is a significant correlation between TP enzyme activity and TP mRNA expression measured by RT-PCR.

This is the first study on *TP* gene expression determined by RT-PCR in epithelial ovarian cancers. *TP* gene expression in stage III–IV was significantly higher compared with stage I–II. Moreover, *TP* gene expression was significantly associated with histological grades. *TP* gene expression of the cases with recurrence showed significantly higher levels compared to those without recurrence. Elevated *TP* gene expression is significantly correlated with reduced survival when examined by univariate analysis. Multivariate analysis, however, demonstrated that *TP* gene expression is not an independent prognostic factor among the clinicopathological parameters studied. It has to be noted that the number of cases was limited. This molecular evaluation indicates that *TP* gene expression level may play one of the key roles in the biology of ovarian epithelial cancer and define a more aggressive tumour phenotype. Lane 2 in Figure 1 presents the results of RT-PCR in patient of stage I (serous cystadenocarcinoma) with primary colorectal cancer who died 2 months after operation. The *TP* gene expression was remarkably high at 4.77. The *TP* gene expression in this primary ovarian cancer tissue might reflect its poor prognosis, although the clinical stage of ovarian cancer was early.

 *© Cancer Research Campaign 1999*

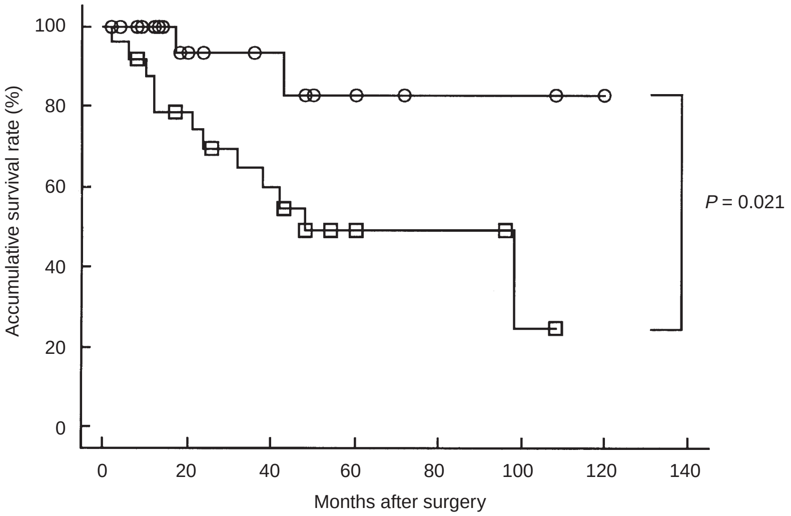

**Figure 6** Comparison of survival between groups with high thymidine phosphorylase (*TP*) gene expression (□; ≥ 0.93; *n* = 25) and low *TP* gene expression (○ < 0.93; *n* = 22) according to the Kaplan–Meier method

The amino acid sequence for TP does not encode a signal peptide, and TP is not a classic paracrine growth factor in this regard (Miyadera et al, 1995). It is suggested that 2′-deoxy-*D*-ribose-1-phosphate, the catalytic product of thymidine by TP, is responsible for various biological activities of TP (Haraguchi et al, 1994). It is also possible that TP is released by a non-classical pathway. TP positivity assessed by immunohistochemistry is associated with microvessel count and prognosis in various cancers (Maeda et al, 1996; O'Brien et al, 1996; Takebayashi et al, 1996; Giatromanolaki et al, 1997). However, TP expression is an independent prognostic factor even after adjustment for microvessel count in tumour tissues (Takebayashi et al, 1996). TP may have effects other than angiogenic activity concerned with the tumour progression. Recently, Takebayashi et al (1997) reported that *TP* is induced by hypoxia in KB 3-1 cells that have no endogenous TP activity and that hypoxia-resistant subclone KB-HR3 cells express *TP*. Furthermore, KPE-3 cells which are transfected with *TP* cDNA are resistant to apoptosis induced by hypoxia (Takebayashi et al, 1997). These results indicate that the hypoxia-mediated *TP* gene induction and selection of TP-expressing cells play an important role in progression of many solid tumours. Further study is needed to assess how *TP* gene expression is associated with unfavourable prognosis of ovarian epithelial cancer.

TP catalyses formation of 5-fluorouracil (5-FU) from its prodrug, 5′-deoxy-5-fluorouridine (5′-DFUR) (Kono et al, 1983). Furthermore, toxicity of the active drug 5-FU may be enhanced by changing into 5-fluoro-2′-deoxyuridine through transfer of 2′-deoxy-*D*-ribose-1-phosphate. These pyrimidine antagonists can ultimately inhibit the synthesis of DNA (Zimmerman and Seidenberg, 1964). It has also been reported that 5-FU can induce apoptosis in some cancer cells (Szepeshazi et al, 1991). Increased sensitivity to 5′-DFUR has been demonstrated in vitro by transfection of the *TP* gene into the MCF-7 breast carcinoma cell line (Patterson et al, 1995). Elevated expression of TP in tumour cells suggests that 5′-DFUR would be differentially activated in tumour cells (O'Brien et al, 1996). It is noted that preoperative chemotherapy with 5′-DFUR is useful for reducing minute cancer foci and microscopic metastatic lesions in gastric cancer (Kumagai et al, 1993). Consequently, it is possible that treatment with 5′-DFUR might be highly selective for the inhibition of tumour progression in epithelial ovarian cancer with high *TP* gene expression. Assessment of *TP* gene expression should be taken into account of randomized trial evaluating the role of adjuvant chemotherapy in epithelial ovarian cancer.

**Table 2** Univariate analysis of survival using the log-rank test

| Variables | *P*-value |
|---|---|
| FIGO stage<br>I–II (*n* = 22) vs III–IV (*n* = 25) | 0.001 |
| Residual disease<br>≤ 2 cm (*n* = 34) vs > 2 cm (*n* = 13) | 0.006 |
| Histology<br>Serous (*n* = 21) vs others (*n* = 26) | 0.647 |
| Grade<br>Others (*n* = 34) vs poorly (*n* = 13) | 0.048 |
| *TP* gene expression<br>< 0.93 (*n* = 22) vs ≥ 0.93 (*n* = 25) | 0.021 |

TP, thymidine phosphorylase.

**Table 3** Multivariate analysis of survival using Cox's proportional hazard model

| Variables | Hazard ratio | 95% CI | *P*-value |
|---|---|---|---|
| FIGO stage<br>I–II (*n* = 22) vs III–IV (*n* = 25) | 4.19 | 1.1–130.4 | 0.04 |
| Residual disease<br>≤ 2 cm (*n* = 34) vs > 2 cm (*n* = 13) | 1.15 | 0.6–6.5 | 0.284 |
| Grade<br>Others (*n* = 34) vs poorly (*n* = 13) | 3.00 | 0.8–7.9 | 0.083 |
| *TP* gene expression<br>< 0.93 (*n* = 22) vs ≥ 0.93 (*n* = 25) | 0.91 | 0.5–13.8 | 0.34 |

TP, thymidine phosphorylase.

### ACKNOWLEDGEMENTS

The work of KH is supported by a grant from Shimane Medical University Education and Research Foundation.

### REFERENCES

Arao S, Suwa H, Mandai M, Tashiro H, Miyazaki K, Okamura H, Nomura H, Hiai H and Fukumoto M (1994) Expression of multidrug resistance gene and localization of P-glycoprotein in human primary ovarian cancer. *Cancer Res* **54**: 1355–1359

Bourne TH, Campbell S, Reynolds KM, Whitehead MI, Hampson J, Royston P, Crayford TJB and Collins WP (1993) Screening for early familial ovarian cancer with transvaginal ultrasonography and colour blood flow imaging. *Br J Med* **306**: 1025–1029

Cannistra as (1993) Cancer of the ovary. *N Engl J Med* **329**: 1550–1559

Cox DR (1972) Regression models and life tables. *J R Stat Soc B* **31**: 1333–1335

Eisenstein BI (1990) The polymerase chain reaction: a new method of using molecular genetics for medical diagnosis. *N Engl J Med* **322**: 181–183

Fox SB, Westwood M, Moghaddam A, Comley M, Turley H, Whitehouse RM, Bicknell R, Gatter KC and Harris AL (1996) The angiogenic factor platelet-derived endothelial cell growth factor/thymidine phosphorylase is up-regulated in breast cancer epithelium and endothelium. *Br J Cancer* **73**: 275–280

Furukawa T, Yoshimura A, Sumizawa T, Haraguchi M, Akiyama S, Fukui K, Ishizawa M and Yamada Y (1992) Angiogenic factor. *Nature* **356**: 668

Giatromanolaki A, Koukourakis MI, Comley M, Kaklamanis L, Turley H, O'Byrne K, Harris AL and Gatter KC (1997) Platelet-derived endothelial cell growth factor (thymidine phosphorylase) expression in lung cancer. *J Pathol* **181**: 196–199

Griffiths CT and Parker L (1986) Cancer of the ovary. In *Gynaecologic Oncology*, Knapp RC and Berkowitz RS (ed), pp. 313–375. Macmillan: New York

Hagiwara K, Stenman G, Honda H, Sahlin P, Andersson A, Miyazono K, Heldin C-H, Ishikawa F and Takaku F (1991) Organization and chromosomal localization of the human platelet-derived endothelial cell growth factor gene. *Mol Cell Biol* **11**: 2125–2132

Haraguchi M, Miyadera K, Uemura K, Sumizawa T, Furukawa T, Yamada K, Akiyama S and Yamada Y (1994) Angiogenic activity of enzymes. *Nature* **368**: 198

Hata K, Hata T and Kitao M (1995) Intratumoral peak systolic velocity as a new possible predictor for detection of adnexal malignancy. *Am J Obstet Gynecol* **172**: 1496–1500

Hata K, Hata T and Collins WP (1997) Association of thymidine phosphorylase concentration with ultrasound-derived indices of blood flow in ovarian masses. *Cancer* **80**: 1079–1084

International Federation of Gynecology and Obstetrics (FIGO) (1987) Changes in definitions of clinical staging for carcinoma of the cervix and ovary. *Am J Obstet Gynecol* **156**: 263–264

Ishikawa F, Miyadera K, Hellman U, Drexler H, Hagiwara K, Usuki K, Takaku F, Risau W and Heldin CH (1989) Identification of angiogenic activity and the cloning and expression of platelet-derived endothelial cell growth factor. *Nature* **338**: 557–562

Kono A, Hara Y, Sugata S, Karube Y, Matsushima Y and Ishitsuka H (1983) Activation of 5′-deoxy-5-fluorouridine by thymidine phosphorylase in human tumors. *Chem Pharma Bull* **31**: 175–178

Kosary CL (1994) FIGO stage, histologic grade, age, and race as prognostic factors in determining survival for cancers of the female gynecological system: an analysis of 1973–87 SEER cases of cancers of the endometrium, cervix, ovary, vulva and vagina. *Semin Surg Oncol* **10**: 31–46

Kumagai K, Yasui A, Nishida Y, Masuo K and Yoshitoshi A (1993) The significance of preoperative chemotherapy for early gastric carcinoma. *Surgery Today* **23**: 875–879

Maeda K, Chung Y-S, Ogawa Y, Takatsuka T, Kang S-M, Ogawa M, Sawada T and Onoda N (1996) Thymidine phosphorylase/platelet-derived endothelial cell growth factor expression associated with hepatic metastasis in gastric carcinoma. *Br J Cancer* **73**: 884–888

Mimori K, Mori M, Shiraishi T, Haraguchi M, Ueo H and Akiyoshi T (1997) Clinical significance of pyrimidine nucleoside phosphorylase in colorectal cancer. *Int J Oncol* **10**: 493–496

Miyadera K, Sumizawa T, Haraguchi M, Yoshida H, Konstanty W, Yanada Y and Akiyama Y (1995) Role of thymidine phosphorylase activity in the angiogenic effect of platelet-derived endothelial cell growth factor/thymidine phosphorylase. *Cancer Res* **55**: 1687–1690

Moghaddam A and Bicknell R (1992) Expression of platelet-derived endothelial cell growth factor in *Escherichia coli* and confirmation of its thymidine phosphorylase activity. *Biochemistry* **31**: 12141–12146

O'Brien TS, Fox SB, Dickinson AJ, Turley H, Westwood M, Moghaddam A, Gatter KC, Bicknell R and Harris AL (1996) Expression of the angiogenic factor thymidine phosphorylase/platelet derived endothelial cell growth factor in primary bladder cancers. *Cancer Res* **56**: 4799–4804

Patterson AV, Zhang H, Moghaddam A, Bickell R, Talbot DC, Stratford IJ and Harris AL (1995) Increased sensitivity to the prodrug 5′-deoxy-5-fluorouridine and modulation of 5-fluoro-2′-deoxyuridine sensitivity in MCF-7 cells transfected with thymidine phosphorylase. *Br J Cancer* **72**: 699–675

Pauly JL, Schuller MG, Zelcer AA, Kriss TA, Gore SS and Germain MJ (1997) Identification and comparative analysis of thymidine phosphorylase in the plasma of healthy subjects and cancer patients. *J Natl Cancer Inst* **58**: 1587–1590

Reynolds K, Farzaneh F, Collins WP, Campbell S, Bourne TH, Lawton F, Moghaddam A, Harris AL and Bickell R (1994) Association of ovarian malignancy with expression of platelet-derived endothelial cell growth factor. *J Natl Cancer Inst* **86**: 1234–1238

Scholl SM, Bascou CH, Mosseri V, Olivares R, Magdelenat H, Dorval T, Palangie T, Validire P, Pouillart P and Stanley ER (1994) Circulating levels of colony-stimulating factor 1 as a prognostic indicator in 82 patients with epithelial ovarian cancer. *Br J Cancer* **69**: 342–346

Szepeshazi K, Lapis K and Scally V (1991) Effect of combination treatment with analogs of luteinizing hormone-releasing hormone (LH-RH) or somatostatin and 5-fluorouracil on pancreatic in hamsters. *Int J Cancer* **49**: 260–266

Takebayashi Y, Akiyama S, Akiba S, Yamada K, Miyadera K, Sumizawa T, Yamada Y, Murata F and Aikou T (1996) Clinicopathologic and prognostic significance of an angiogenic factor, thymidine phosphorylase, in human colorectal carcinoma. *J Natl Cancer Inst* **88**: 1110–1117

Takebayashi Y, Akiyama S, Tani A, Haraguchi M, Furukawa T, Miyadera K, Yamada Y and Aikou T (1997) Induction of thymidine phosphorylase by hypoxia and relationship between expression of thymidine phosphorylase and apoptosis. *Proc Annu Am Assoc Cancer Res* **38**: 48

Yoshimura A, Kuwazuru Y, Furukawa T, Yoshida H, Yamada K and Akiyama S (1990) Purification and tissue distribution of human thymidine phosphorylase: high expression in lymphocytes, reticulocytes and tumors. *Biochim Biophys Acta* **1034**: 107–113

Zimmerman M and Seidenberg J (1964) Deoxyribosyl transfer. Thymidine phosphorylase and nucleoside deoxyribosyltransferase in normal and malignant tissue. *J Biol Chem* **230**: 2618–2621

 *© Cancer Research Campaign 1999*
